# Dental fluorosis in populations from Chiang Mai, Thailand with different fluoride exposures – Paper 1: assessing fluorosis risk, predictors of fluorosis and the potential role of food preparation

**DOI:** 10.1186/1472-6831-12-16

**Published:** 2012-06-21

**Authors:** Michael G McGrady, Roger P Ellwood, Patcharawan Srisilapanan, Narumanas Korwanich, Helen V Worthington, Iain A Pretty

**Affiliations:** 1 School of Dentistry, University of Manchester, Manchester, M13 9PL, England, UK; 2 Colgate Palmolive Dental Health Unit, 3A Skelton House, Lloyd Street North, Manchester, M15 6SH, England, UK; 3 Faculty of Dentistry, Chiang Mai University, Chiang Mai, Thailand

## Abstract

**Background:**

To determine the severity of dental fluorosis in selected populations in Chiang Mai, Thailand with different exposures to fluoride and to explore possible risk indicators for dental fluorosis.

**Methods:**

Subjects were male and female lifetime residents aged 8–13 years. For each child the fluoride content of drinking and cooking water samples were assessed. Digital images were taken of the maxillary central incisors for later blind scoring for TF index (10% repeat scores). Interview data explored previous cooking and drinking water use, exposure to fluoride, infant feeding patterns and oral hygiene practices.

**Results:**

Data from 560 subjects were available for analysis (298 M, 262 F). A weighted kappa of 0.80 was obtained for repeat photographic scores. The prevalence of fluorosis (TF 3+) for subjects consuming drinking and cooking water with a fluoride concentration of <0.9 ppm was 10.2%. For subjects consuming drinking and cooking water >0.9 ppm F the prevalence of fluorosis (TF 3+) rose to 37.3%. Drinking and cooking water at age 3, water used for infant formula and water used for preparing infant food all demonstrated an increase in fluorosis severity with increase in water fluoride level (p < 0.001). The probability estimate for the presentation of aesthetically significant fluorosis was 0.53 for exposure to high fluoride drinking (≥0.9 ppm) and cooking water (≥1.6 ppm).

**Conclusions:**

The consumption of drinking water with fluoride content >0.9 ppm and use of cooking water with fluoride content >1.6 ppm were associated with an increased risk of aesthetically significant dental fluorosis. Fluoride levels in the current drinking and cooking water sources were strongly correlated with fluorosis severity. Further work is needed to explore fluorosis risk in relation to total fluoride intake from all sources including food preparation.

## Background

The benefits of fluoride in the prevention and control of dental caries have been accepted for many years. However, alongside these benefits it is recognized that the ingestion of fluoride during the period of tooth development increases the risk of developing dental fluorosis, a developmental defect seen as hypomineralization of the enamel [1].

The severity of fluorosis is dependent on a number of factors including the level of fluoride ingested and the time period this ingestion takes place [2,3]. Reviews of data generated from water fluoridation and fluoride supplement studies suggest there is a strong linear relationship between the severity of dental fluorosis and the fluoride dose [4,5].

In populations with low or moderate exposure to fluoride through optimally fluoridated community water supplies and fluoridated dentifrices, fluorosis may present as diffuse white lines or opacities of the enamel surface as a result of an increase in the porosity of the fluorotic enamel. However, in populations exposed to higher levels of fluoride for example, high levels of fluoride in groundwater used for cooking and drinking, fluorosis may manifest as more severe hypomineralization with pitting and loss of the surface enamel. Such a population exposed to high levels of fluoride in groundwater exists in Chiang Mai, Thailand. Chiang Mai Province lies in the Chiang Mai Basin in Northern Thailand. Water is fairly abundant in the form of both surface and ground water. In the cities of Chiang Mai, Doi Saket and Mae Rim the domestic water supply is based largely on surface water. The other cities and villages of the province have water supplies that are derived from groundwater sources [6] where the fluoride content has been shown to range between 0 – 16 mg/l [7]. The distribution of groundwater fluoride across the region appears to be linked with geothermal activity and fault lines with the fluoride level dependent upon well depth, however this data is not clearly documented [6,7].

Owing to low awareness of risks of the high fluoride content of the groundwater in the region, endemic dental fluorosis developed in the population [8]. In response to this efforts were made by the Thai government and the Intercountry Centre for Oral Health (ICOH) to educate the population to the risks of excessive fluoride consumption and to defluoridate the water supply [7,9]. In the larger communities this could be achieved by defluoridation of the public water supply through the use of reverse osmosis and experimental studies using nano-filtration [8]. In the smaller villages and communities the use of defluoridators and bone char buckets were introduced. In some areas the continued use of household defluoridators was not successful. This was largely owing to difficulties in replacing filters for ICOH defluoridators that required periodic replacement, a process the ICOH was unable to sustain. As a result the population were advised to use bottled water for drinking. Bottled water is now widely used as the main source for drinking water where defluoridated water cannot be provided [7].

Despite the efforts to defluoridate water sources, a wide range of fluoride levels persist in groundwater sources in Chiang Mai and fluoride intake varies according to the water sources available. This provides a unique opportunity to explore the effects of fluoride on the dentition in particular the dose response between fluoride and resulting dental fluorosis. The objectives of this study were to determine the severity of dental fluorosis in selected populations with different exposures to fluoride and to explore the risk factors and possible predictors associated with dental fluorosis, in particular water use, infant feeding patterns and oral hygiene practices.

## Methods

The protocol for the study was approved by the Human Experimentation Committee, Faculty of Dentistry, Chiang Mai University, Thailand (clearance number 1/2008) Notification was given to the University of Manchester Committee on Ethics on Research on Human Beings.

The study was an observational cross-sectional survey based on a convenience sample population with varying exposures to fluoride and was part of a larger project exploring the detection and quantification of enamel fluorosis.

### Screening and selection of subjects

Subjects were selected with a view to recruiting populations at varying levels of fluoride exposure. The aim was to recruit subjects into approximately six population groups exposed to a range of water fluoride content: <0.01 ppm, 0.5 ppm, 0.75 ppm, 1.00 ppm, 1.5 ppm, 2 + ppm. Subjects were recruited with the aim to obtain equal numbers between the population groups with the pattern of recruitment monitored to reduce imbalance between the population groups. The aim was to recruit approximately 100 subjects in each group. A sample size calculation determined that a continuity corrected *χ*^2^ test with a 0.05 two-sided significance level would have 80% power to detect the difference between a group 1 proportion of 20% and a group 2 proportion of 40% (odds ratio of 2.667) with 91 subjects per group. Schools in the Chiang Mai area were targeted for high, expected levels of cooperation and low population mobility. All parents of children in school year groups covering ages 8 to 13 years old were approached to seek consent for their children to participate. A written consent was obtained from the parents with written and or verbal assent obtained from the children. Eligibility criteria for the study required subjects to be lifelong residents of their particular locality, to be in good general health with both maxillary incisors fully erupted and free from fixed orthodontic appliances.

Water samples were collected from all consented subjects in order to determine fluoride content. Samples for drinking and cooking water were obtained. In communities where a common water supply was used for drinking and/or cooking (such as a village well or municipal supply), analysis of a single water sample was undertaken. Water analysis was carried out by the Science and Technology Service Centre, Chiang Mai University according to an analytical protocol. The fluoride content of the samples was determined using a 4-Star Benchtop pH/ISE meter, Orion Company, Mass, USA. In order to assign the subjects in to groups the data generated from the cooking water were used. This was owing to the fact there was a wider range and variation in the fluoride content of the cooking water compared to the drinking water.

Upon recruitment subjects were assigned a five-digit subject ID number. The first two digits specified the school and the next 3 digits the subject’s individual study number based on the sequence of their recruitment.

### Photographic examination

Recruited subjects had conventional digital images taken of the maxillary central incisors. A lip retractor was used to isolate the teeth and the upper anterior teeth were cleaned with a toothbrush and then dried using a cotton wool roll for a period of one minute. The dried teeth were viewed under indirect natural light (not direct sunlight) Standardized digital images were taken with a Nikon D100 camera with a Micro Nikkor 105 mm lens and a Nikon SB 21 ringflash using only the upper illumination element. Images were captured at an angle of 15 degrees to perpendicular in order to minimize specula reflection with a 1:1 reproduction ratio (life size). None of the images contained any identifying aspects of the subjects face. A photographic log form was completed to enable the digital files to be linked to the unique subject identifier.

The digital photographic images were exported to a computer and transported to the School of Dentistry, The University Manchester, England. The images were then integrated into a graphical user interface that randomized and blinded the images which were then displayed on a 32 inch flat screen monitor under controlled lighting. A consensus score for Thylstrup and Fejerskov Index (TF) [1] was then given for each image by two examiners (R.P.E and M.G.M). This was recorded directly by the interface into a Windows (Microsoft Corp., Seattle, Wash., USA) excel file and imported into the Statistical Package for Social Sciences (SPSS 16.0) for statistical analysis.

### Interview

Each subject and their parent or guardian took part in a structured interview process in their homes with a team of trained interviewers. Information was recorded pertaining to history of residence, school, age and gender. Patterns of water use were also recorded from birth to age three years and current water use for both cooking and drinking e.g. tap, well, ground and bottle (including the brand name). Infant feeding patterns were also investigated such as breast or formula feeding (including the water used for reconstitution) and the types infant foods after weaning, particularly the consumption of rice. The type of water used for the preparation of foods was also noted. Subjects were asked about their oral hygiene practises, when they first started to brush, tooth brushing frequency, brand of dentifrice and whether they swallowed dentifrice. The interview used a combination of close-ended and partially close-ended questions and allowed for validation of some responses. The information from the interview was entered into SPSS and used to verify lifetime residency, age of the subjects and to explore risk indicators for dental fluorosis.

### Data management and analysis

In order to examine the population groups in terms of water fluoride content, frequency distributions of fluoride content were examined for both drinking and cooking water. Appropriate intervals were created according to the frequency distribution of subjects for the fluoride content of the cooking water samples in order to create approximately equal groups. This would attempt to create balanced groups of subjects comparable to the ideals set out at recruitment.

Variables were also created to explore the data with respect to risk factors associated with fluorosis. Interview information on the water source used for drinking and cooking at age three, water used to reconstitute baby formula and water used to prepare infant food were converted into new variables that were comparable to the intervals created for the fluoride content of the current drinking and cooking water from the water sample analysis. Information relating to feeding patterns obtained at interview was converted into a categorical variable: breast feeding alone, formula feeding alone and combination of breast and formula feeding. Variables were also created for the age at which toothbrushing commenced, the frequency of toothbrushing, the fluoride content of toothpaste and gender.

The primary outcome measure for fluorosis was the consensus score from the digital photographs. The basis for this decision was that it was less prone to bias than the clinical score, the examiners were blinded to the probable fluoride exposure and the images were presented in a randomized order. In addition, as the score was a consensus score from two examiners, it would potentially reduce problems associated with examiner personal thresholds related to scoring less severe presentations of fluorosis (TF 1, 2) [10]. Additional variables were created to group TF scores of 4 and above (TF 4+) within the TF scale and a dichotomous variable of TF scores 0–2 and TF scores 3+ to represent presence or absence of aesthetically significant fluorosis [11]. A sample of photographic images were randomly selected and scored again for TF by the examiners in order to assess reproducibility.

A bivariate analysis for each of the risk factors was conducted using ANOVA and *χ*^2^ tests where appropriate. Unadjusted odds ratios were estimated with logistic regression.

A multivariate logistic regression was conducted to identify the explanatory variables considered to be independent indicators of the presentation of aesthetically significant fluorosis (TF score 3+) with a dichotomous TF Index fluorosis score as the dependant variable (TF 0–2, TF 3+). Using a forward stepwise model, variables were included in the model if they were a significant indicator of the presence or absence of aesthetically significant fluorosis. Variables were excluded if there was multi collinearity or if the variable was found not to be a significant indicator aesthetically significant fluorosis.

## Results

**Figure 1:**
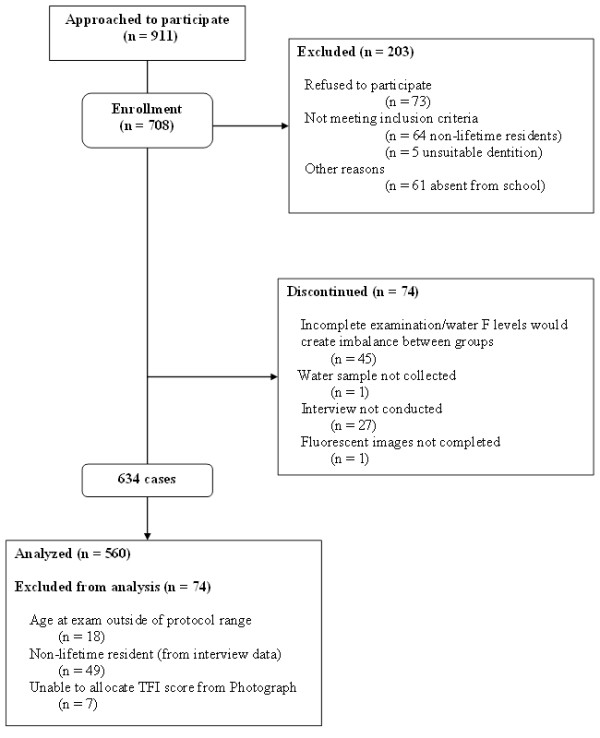
Subject accountability illustrating flow of subjects through each stage of the study.

Nine hundred and eleven (911) subjects from eleven (11) schools were approached to participate in this survey. Seventy three (73) subjects did not provide consent to participate. Eight hundred and thirty eight (838) subjects consented to participate, following screening for suitability seven hundred and eight subjects (708) were enrolled onto the study. Subject accountability is detailed in Figure 1. Photographic examinations and interviews took place between December 2007 and September 2008. Six hundred and thirty four subjects (634) were included in the study following completion of photographic examinations and interviews. Subjects were excluded from the examinations if they were deemed to be non-lifetime residents, had unsuitable dentition or if inclusion based on the water fluoride analysis results would have created imbalance in the population groups. Additional subjects were removed from the analysis during data checking and are described in Figure 1. Subjects were excluded if information from the interview conflicted with demographic data relating to lifetime residency and age at time of examination. Subjects were also excluded if the upper maxillary teeth could not be ascribed a TF score from the photographs – this would have resulted from the presence of restorations, loss of tooth tissue owing to trauma and presence of extrinsic stain. In total five hundred and sixty (560) subjects were available for analysis. There were 298 males (mean age at exam 10.44, range 8–13) and 262 females (mean age at exam 10.48, range 8–13).

Reproducibility for the photographic image scores was performed on sixty five (65) randomly selected images five (5) months after the original assessments. A weighted Kappa value of 0.80 was obtained (SE 0.05, 95% CI 0.71, 0.89) demonstrating good agreement with the examiners using the full range for TF scores for the images presented. The repeat consensus score for TF was never more than one unit different to the initial assessment.

Descriptive statistics are presented in Table 1 for the distribution of each independent variable for each of the TF score categories. The data illustrates as the mean values of fluoride concentration in current drinking and current cooking water increase the fluorosis severity increases. For subjects with a TF score of 0 the mean fluoride concentration for drinking and cooking water was 0.35 ppm (SD 0.37) and 0.65 ppm (SD 0.84) respectively. For subjects presenting with TF scores of 4 or higher the mean fluoride content increased to 0.83 ppm (SD 0.90) and 2.23 ppm (SD 1.52) respectively.

**Table 1:** Distribution of independent variables for each fluorosis category

	**TF 0**			**TF 1**			**TF 2**			**TF 3**			**TF 4+**			**Row total**
	**N**	**Mean**	**(SD)**	**N**	**Mean**	**(SD)**	**N**	**Mean**	**(SD)**	**N**	**Mean**	**(SD)**	**N**	**Mean**	**(SD)**	
**Water Continuous Data**																
Drinking water ppm F	163	0.35	(0.37)	209	0.37	(0.39)	94	0.47	(0.40)	51	0.50	(0.44)	43	0.83	(0.90)	560
Cooking water ppm F (N=560)	163	0.65	(0.84)	209	1.04	(1.02)	94	1.10	(0.87)	51	1.12	(0.93)	43	2.23	(1.52)	560
	**N**	**(%)**		**N**	**(%)**		**N**	**(%)**		**N**	**(%)**		**N**	**(%)**		**Row Total**
**Water Interval Data (ppmF)**																
Drinking water: <0.20	79	(48)		82	(39)		23	(24)		15	(29)		11	(26)		210
0.2 to 0.59	55	(34)		85	(41)		45	(48)		21	(41)		12	(28)		218
0.6 to 0.89	18	(11)		22	(10)		13	(14)		6	(12)		4	(9)		63
0.9+	11	(7)		20	(10		13	(14)		9	(18)		16	(37)		69
Total	163	(100)		209	(100)		94	(100)		51	(100)		43	(100)		560
Cooking water: <0.20	52	(32)		39	(19)		5	(5)		6	(12)		1	(2)		103
0.2 to 0.59	44	(27)		36	(17)		21	(22)		7	(14)		3	(7)		111
0.6 to 0.89	37	(23)		44	(21)		25	(27)		12	(23)		5	(12)		123
0.9 to 1.59	18	(11)		49	(23)		21	(22)		12	(23)		11	(26)		111
1.6+	12	(7)		41	(20)		22	(23)		14	(28)		23	(53)		112
Total	163	(100)		209	(100)		94	(100)		51	(100)		43	(100)		560
Drinking water: <0.20	67	(41)		63	(16)		15	(16)		10	(20)		10	(23)		165
(Age 3) 0.2 to 0.59	52	(32)		76	(43)		40	(43)		13	(26)		10	(23)		191
0.6+	44	(27)		67	(42)		39	(41)		28	(54)		23	(54)		201
Total	163	(100)		206	(100)		94	(100)		51	(100)		43	(100)		557
Cooking water: <0.20	53	(33)		41	(20)		9	(10)		7	(14)		4	(14)		114
(Age 3) 0.2 to 0.59	49	(30)		44	(21)		25	(27)		8	(16)		7	(25)		133
0.6 to 0.89	35	(21)		44	(21)		25	(27)		12	(23)		1	(4)		117
0.9 to 1.59	17	(10)		41	(20)		19	(20)		11	(22)		7	(25)		95
1.6+	9	(6)		37	(18)		16	(17)		13	(25)		9	(32)		84
Total	163	(100)		207	(100)		94	(100)		51	(100)		28	(100)		543
Water formula: <0.20	36	(36)		39	(29)		13	(19)		6	(20)		4	(14)		98
0.2 to 0.59	32	(32)		50	(37)		29	(41)		9	(29)		7	(25)		127
0.6 to 0.89	19	(19)		18	(13)		15	(21)		7	(23)		1	(4)		60
0.9 to 1.59	8	(8)		17	(13)		7	(10)		5	(16)		7	(25)		44
1.6+	4	(4)		11	(8)		6	(9)		4	(13)		9	(32)		34
Total	99	(100)		135	(100)		70	(100)		31	(100)		28	(100)		363
Water Infant Food: <0.20	58	(37)		51	(25)		10	(11)		11	(21)		1	(2)		131
0.2 to 0.59	47	(30)		50	(24)		29	(31)		10	(20)		6	(14)		142
0.6 to 0.89	28	(18)		38	(19)		23	(25)		10	(20)		4	(9)		103
0.9 to 1.59	14	(9)		34	(17)		16	(17)		10	(20)		10	(23)		84
1.6+	9	(6)		31	(15)		15	(16)		10	(20)		22	(51)		87
Total	156	(100)		204	(100)		93	(100)		51	(100)		43	(100)		547
	**N**	**(%)**		**N**	**(%)**		**N**	**(%)**		**N**	**(%)**		**N**	**(%)**		**Row Total**
**Oral Hygiene Practices**																
Age toothbrush start: 4 years+	20	(13)		31	(15)		14	(15)		7	(14)		4	(10)		76
3-4 years	43	(28)		44	(22)		25	(27)		13	(26)		13	(32)		138
2-3 years	48	(31)		67	(33)		34	(37)		14	(28)		15	(38)		178
1-2 years	35	(23)		54	(26)		17	(19)		12	(24)		8	(20)		126
0-1 year	8	(5)		9	(4)		2	(2)		4	(8)		0	(0)		23
Total	154	(100)		205	(100)		92	(100)		50	(100)		40	(100)		541
Toothbrushing freq:1/day	45	(28)		40	(19)		23	(24)		13	(25)		9	(21)		130
2	99	(61)		145	(69)		60	(64)		30	(59)		26	(60)		360
3+	19	(12)		24	(12)		11	(12)		8	(16)		8	(19)		70
Total	163	(100)		209	(100)		94	(100)		51	(100)		43	(100)		560
F content paste: < 1000 ppm)	13	(8)		24	(12)		7	(7)		5	(10)		10	(23)		59
1000 ppmF	150	(92)		185	(88)		87	(93)		46	(90)		33	(77)		501
Total	163	(100)		209	(100)		94	(100)		51	(100)		43	(100)		560
**Other Variables**																
	**N**	**(%)**		**N**	**(%)**		**N**	**(%)**		**N**	**(%)**		**N**	**(%)**		**Row Total**
Feeding pattern: Breast alone	47	(32)		58	(30)		18	(21)		20	(40)		13	(32)		156
Breast & formula	88	(59)		119	(61)		55	(66)		24	(48)		19	(46)		305
Formula only	14	(9)		17	(9)		11	(13)		6	(12)		9	(22)		57
Total	149	(100)		194	(100)		84	(100)		50	(100)		41	(100)		518
Gender: male	83	(51)		118	(57)		46	(49)		27	(53)		24	(56)		298
female	80	(49)		91	(43)		48	(51)		24	(47)		19	(44)		262
Total	163	(100)		209	(100)		94	(100)		51	(100)		43	(100)		560

The results of the water fluoride analysis provided a more complex range of data than anticipated in the study planning. This necessitated the creation of arbitrary water intervals based upon the distribution of the water fluoride data. Allocation of subjects to water fluoride intervals based on the frequency distribution of cooking water fluoride content resulted in the creation of five (5) intervals cooking water and four (4) corresponding intervals for drinking water. The details of these intervals and the distribution of subjects are illustrated in Table 1.

The variables associated with water interval data demonstrated as the fluoride content of the water increased, greater numbers of subjects presented with fluorosis of increasing severity. This was true of the interval data for current drinking and cooking water derived from the water analysis data and also for the variables created from the interview data. These variables were drinking and cooking water at age three (Drinking water age 3, Cooking water age 3), water used for preparing infant food (Water Infant Food) and water used to reconstitute infant formula (Water formula). This pattern was less clear for the variables relating to oral hygiene practices. Insufficient reliable data were available for the reported history of swallowed dentifrice and was excluded from the analysis. This was largely due to a lack of recall. Where this data were available exploratory analysis suggested no pattern associated with the presentation of fluorosis in this population.

There appeared to be no clear pattern in this population between the severity of fluorosis presentation, the age at which tooth brushing commenced, the frequency of toothbrushing and the fluoride content of toothpaste. This was also true of infant feeding practises.

The overall prevalence of fluorosis in the study population was 70.9% (Table 2) with a prevalence of aesthetically significant fluorosis (TF 3+) of 16.8%. To evaluate the effect of differing fluoride levels of both drinking and cooking water on fluorosis severity, data were combined into <0.9 ppm fluoride and >0.9 ppm fluoride categories i.e. grouping together water intervals to produce dichotomous variables. The rationale for these arbitrary cut offs was based upon both the study water interval cut offs (derived from data distribution) and the approximation to historical values for community water supply fluoride levels for caries prevention incorporating climate and fluorosis risk [12-14]. The prevalence of fluorosis among subjects consuming drinking and cooking water <0.9 ppm fluoride was 60.6% (10.1% for TF 3+). The prevalence of fluorosis among subjects consuming drinking and cooking water >0.9 ppm fluoride was 85.1% (16.8% for TF 3+).

**Table 2:** Prevalence data for fluorosis (accounting for combined drinking and cooking water sources)

**Combined water sources**	**Fluorosis prevalence (n)**				
	**TF Score**				
	**0**	**1+**	**2+**	**3+**	**4+**
Drinking water <0.9 ppm F	39.4% (132)	60.6% (119)	25.1% (50)	10.1% (25)	2.7% (9)
Cooking water <0.9 ppm F					
*Drinking water >0.9 ppm F*	(1)*	(0)*	(1)*	(0)*	(0)*
*Cooking water <0.9 ppm F*					
Drinking water <0.9 ppm F	12.8% (20)	87.2% (70)	42.3% (31)	22.4% (17)	11.5% (18)
Cooking water >0.9 ppm F					
Drinking water >0.9 ppm F	14.9% (10)	85.1% (20)	55.2% (12)	37.3% (9)	23.9% (16)
Cooking water >0.9 ppm F					
**Total study population**	29.1% (163)	70.9% (209)	33.6% (91)	16.8% (54)	7.7% (43)

*prevalence data not calculated owing to low numbers in cells.

Results of the bivariate analysis of each explanatory variable and TF score are presented in Table 3.This was for both the TF score (5 categories) and a dichotomous variable based on the presence or absence of aesthetically significant fluorosis (TF 0–2 versus TF 3+).

**Table 3:** Bi-variate analysis of each risk factor and TF score (as five categories and dichotomised)

**Explanatory Variables : Water Continuous Data obtained by water sample analysis (ppm F)**	**TF score (5 categories)**			**TF 0–2 versus 3+**		
	**ANOVA**			**Binary Logistic Regression**		
	**F-ratio**	**df**	**p-value**	**Odds ratio**	**p-value**	**(95% CI)**
Drinking water	11.31	4, 555	<0.001	2.71*	<0.001	(1.75, 4.18)
Cooking water	22.27	4, 555	<0.001	1.67*	<0.001	(1.39, 2.01)
**Explanatory Variables: Water Interval Data (ppm F)**	**Cross Tabulations**			**Binary Logistic Regression**		
	***χ***^**2**^	**df**	**p-value**	**Odds ratio**	**p-value**	**(95% CI)**
Drinking water (ref <0.20)	45.97	12	<0.001			
0.2 to 0.59				1.26	0.41	(0.73, 2.20)
0.6 to 0.89				1.33	0.47	(0.61, 2.94)
0.9+				4.02	<0.001	(2.12, 7.63)
Cooking water (ref <0.20)	93.33	16	<0.001			
0.2 to 0.59				1.36	0.55	(0.50, 3.71)
0.6 to 0.89				2.20	0.94	(0.87, 5.33)
0.9 to 1.59				3.58	0.005	(1.47, 8.77)
1.6+				6.77	<0.001	(2.86, 16.02)
Drinking water (ref <0.20)	34.62	8	<0.001			
(Age 3) 0.2 to 0.59				0.99	0.98	(0.52, 1.88)
0.6+				2.47	0.002	(1.40, 4.34)
Cooking water (ref <0.20)	83.582	16	<0.001			
(Age 3) 0.2 to 0.59				1.16	0.74	(0.47, 2.87)
0.6 to 0.89				1.87	0.15	(0.80, 4.39)
0.9 to 1.59				3.27	0.005	(1.43, 7.50)
1.6+				6.28	<0.001	(2.82, 13.96)
Water formula (ref <0.20)	40.74	16	= 0.001			
0.2 to 0.59				1.27	0.58	(0.55, 2.93)
0.6 to 0.89				1.35	0.55	(0.50, 3.65)
0.9 to 1.59				3.30	0.12	(1.30, 8.38)
1.6+				5.45	<0.001	(2.10, 14.11)
Water infant food (ref <0.20)	87.13	16	<0.001			
0.2 to 0.59				1.26	0.57	(0.57, 2.77)
0.6 to 0.89				1.56	0.29	(0.69, 3.54)
0.9 to 1.59				3.10	0.004	(1.42, 6.74)
1.6+				5.77	<0.001	(2.76, 12.05)
**Explanatory Variables : Oral Hygiene Practices**	***χ***^**2**^	**df**	**p-value**	**Odds ratio**	**p-value**	**(95% CI)**
Age toothbrush starts (ref 4 years+)	11.18	16	0.80			
3-4 years				1.37	0.42	(0.64, 2.96)
2-3 years				1.15	0.72	(0.50, 2.45)
1-2 years				1.12	0.79	(0.54, 2.48)
0-1 year				1.24	0.73	(0.36, 4.36)
Toothbrushing frequency (ref once per day)	6.63	8	0.58			
2				0.90	0.331	(0.53, 1.55)
3+				1.46	0.309	(0.71, 3.00)
Fluoride content of paste (ref < 1000 ppm)	9.69	4	0.46			
1000 ppmF				0.55	0.06	(0.29, 1.04)
**Other explanatory variables**	***χ***^**2**^	**df**	**p-value**	**Odds ratio**	**p-value**	**(95% CI)**
Feeding pattern (ref Breast alone)	12.87	8	0.12			
Breast & formula				0.61	0.61	(0.37, 1.01)
Formula only				1.33	0.43	(0.66, 2.69)
Gender (ref male)	2.04	4	0.729			
female				0.95	0.83	(0.61, 1.48)

* odds ratios reported, but residuals strongly related to the fluoride levels for both variables.

Variables for fluoride content of current drinking and cooking water (obtained from water analysis), content of cooking and drinking water at age 3 (obtained from interview data), water used for infant formula, cooking infant food (all obtained from interview data) were all found to have a significant association with the presentation of fluorosis. This was reflected in the unadjusted odds ratios. For current drinking water interval data the odds ratio for the presentation of aesthetically significant fluorosis was 4.02 (p < 0.001; 95% CI 2.12, 7.63) for subjects consuming drinking water with a fluoride content ≥0.9 ppm relative to subjects consuming drinking water <0.2 ppm fluoride. For current cooking water interval data the odds ratio for the presentation of aesthetically significant fluorosis was 6.77 (p < 0.001; 95% CI 2.86, 16.02) for subjects using cooking water with a fluoride content ≥1.6 ppm relative to subjects using cooking water <0.2 ppm fluoride.

All of the remaining explanatory variables demonstrated no significant association with the presentation of fluorosis. The variables for toothbrushing frequency, age at which toothbrushing commenced and infant feeding pattern were found not to have significant association with fluorosis score in this population. The one exception was fluoride content of toothpaste which actually demonstrated a decrease in fluorosis with fluoride content of 1000 ppm when compared to fluoride content <1000 ppm. However, this did not achieve statistical significance (p = 0.06).

When all of the variables were entered into a forward stepwise regression analysis the model yielded contained two variables that were the best indicators for the presence of aesthetically significant fluorosis: the fluoride content of the current drinking and current cooking water. However, the attempt to fit a logistic regression model with the continuous variables resulted in the assumptions underlying logistic regression not being upheld. The residuals were strongly related to the fluoride levels for both variables and increased as the water fluoride level increased.

The data were exported to Stata (release 11, StataCorp, TX, USA) for further analysis. A logistic regression model for dichotomised threshold of fluorosis (presence or absence of aesthetically significant fluorosis) with the independent variable for the current drinking water fluoride content coded as water interval data were fitted. The fit improved significantly when the water interval data for current cooking water was added to the model (Likelihood-ratio test, LR *χ*^2^ (4df) = 30.09, <0.001). The clustering of the children within schools was also taken into account by using the robust standard errors. This data is presented in Table 4. The odds ratio for the presentation of aesthetically significant fluorosis was 3.34 (robust SE 1.22; 95%CI 1.52, 7.04) for subjects consuming drinking water with a fluoride content equal to or greater than 0.9pmm relative to drinking water consumption with less than 0.2 ppm fluoride. The odds ratio for the presentation of aesthetically significant fluorosis was 5.54 (robust SE 3.01; 95%CI 1.91, 16.04) for subjects consuming cooking water with fluoride content equal to or greater than 1.6 ppm relative to cooking water consumption with less than 0.2 ppm fluoride.

**Table 4:** Final Logistic regression model for predicting presence or absence of aesthetically significant fluorosis (TF3+), including the clustering of the children in 11 schools

	**Odds ratio (Robust SE)**	**p-value**	**(95% CI)**
Drinking water (ppm):		0.019	
0.20 to 0.59	1.35 (0.60)	0.50	(0.66, 2.33)
0.60 to 0.89	1.61 (0.64)	0.23	(0.61, 3.66)
0.9+	3.34 (1.22)	0.001	(1.52, 7.04)
Cooking water (ppm):		<0.001	
0.20 to 0.59	1.21 (0.74)	0.75	(0.37, 4.03)
0.60 to 0.89	1.85 (0.94)	0.22	(0.69, 5.01)
0.90 to 1.59	1.85 (1.07)	0.29	(0.59, 5.77)
1.6+	5.54 (3.01)	0.002	(1.91, 16.04)

N = 560.

The presence of any interaction between the fluoride level in the drinking and cooking water was investigated. The overall p-value for this was 0.28 and many of the categories were excluded due to collinearity and small numbers of subjects. Table 5 presents the probability estimates and numbers of subjects for each category when these two variables are cross classified. It can be seen the probability of aesthetically significant fluorosis rises to 0.53 if there is exposure to high levels of fluoride in both drinking (≥0.9 ppm) and cooking water (≥1.6pmm). There was no evidence of an interaction from the probabilities shown here.

**Table 5:** Cross-tabulation of the predicted probabilities of having aesthetically significant fluorosis (TF3+) for the fluoride levels in the drinking and cooking water (number of subjects)

	**Cooking water (ppm)**					
		**0 to 0.19**	**0.20 to 0.59**	**0.60 to 0.89**	**0.90 to 1.59**	**1.60 +**
**Drinking Water (ppm)**	**0 to 0.19**	0.06 (103)	0.07 (103)	0.10 (32)	0.10 (31)	0.25 (51)
	**0.20 to 0.59**	0.08 (15)	0.09 (96)	0.13 (46)	0.13 (29)	0.31 (51)
	**0.60 to 0.89**	0.09 (10)	0.11 (3)	0.15 (52)	0.15 (2)	0.35 (4)
	**0.9+**	-	0.20 (1)	0.28 (2)	0.27 (57)	0.53 (18)

## Discussion

The effects of endemic fluorosis in certain regions of Thailand have been known for some time. It is a problem not unique to Thailand, as many areas of Africa and Asia have similar issues with excessive fluoride consumption resulting in efforts to remove excessive fluoride from drinking water employing various techniques such as coagulation-precipitation, adsorption, ion-exchange and more recently nano-filtration [15-17]. The different techniques are associated with varying levels of effectiveness linked to logistical and financial considerations. The use of reverse osmosis, nano-filtration and bone char defluoridators has been reported in Thailand along with the difficulties associated with the sustainability of such schemes. The use of cheaper alternative methods of defluoridation such as the Nalgonda Technique (popular in parts of India) utilizing alumina, lime and bleach to coagulate and precipitate fluoride from the water supply may not a viable option in this region of Thailand as the sludge produced becomes a waste substance that is difficult to manage. There are also questions regarding the efficacy and sustainability of this technique [16].

In general, the main objective is to provide a community water supply that is safe to drink. In the case of communities supplied by treated surface water the fluoride content of the water supply is lower than treated water from groundwater sources. Nevertheless, the efforts of the Thai government and the ICOH on educating the population with respect to the risks of consuming groundwater with high fluoride content have been successful, although as this was a cross-sectional survey it is not possible to measure the impact of these changes in practice. However, when comparing a subject’s drinking water with their cooking water, 53.2% of subjects consumed drinking water with lower fluoride content. Only 11.4% of subjects consumed drinking water with a higher fluoride content than their cooking water. Where this was the case it was generally as a result of consuming bottled water with low fluoride content while cooking with de-fluoridated or fluoride free community water. When this scenario was cross-tabulated with the TF scores only one subject had a TF score of >3. This suggests the message over the level fluoride in drinking water has been received with some success.

The data suggests in this population the use of cooking water with high levels of fluoride is associated with an increased risk of developing aesthetically significant dental fluorosis. It could be argued the use of data for current drinking and cooking water is inappropriate when assessing the fluorosis status of the subjects. A more appropriate measure would be the use of data obtained from fluoride content of water consumed from birth as part of an assessment of total fluoride intake. An attempt to address this issue was carried out by using data obtained from interview, with the creation of variables of water use at the age of three years comparable to the water intervals derived from the current water sources. Inevitably there would be an element of variance in these variables and also an element of recall bias from interview data.

Nevertheless, the results suggest the best indicators for the presence of aesthetically significant fluorosis were the variables related to current drinking and cooking water. All variables derived from the interview data were excluded from the model during regression analysis (although this was not always necessarily due to a lack of statistical significance but due to the existence of collinearity). Furthermore, the subjects were lifetime residents and the likelihood there had been a change in water supply (particularly cooking water) was low. The spurious result obtained for the fluoride content of toothpaste may be explained by exploring the water fluoride content of the subjects with high TF scores. Without exception these subjects resided in areas with high water fluoride content and were probably advised to use low or non-fluoridated toothpaste. This may also explain why the available data on the swallowing of dentifrice suggested no pattern of association with fluorosis presentation in this population. It is clear from the data in this population there are several factors of great significance that may have a greater impact than the fluoride content of toothpaste and the age at which toothbrushing commences when assessing fluorosis risk.

Several risk factors to fluorosis in this study have not been fully explored or have been found to be non-significant within this population. In the latter case this is more likely to be owing to the lack of robust data or as a result of the implementation of policies to address endemic fluorosis (bottled drinking water, low fluoride toothpaste). This situation arose largely as this study was a cross-sectional survey.

Information relating to infant feeding patterns is essential in assessing fluorosis risk and reliable data for the duration of breast feeding was not available. It was not possible to establish the presence of any protective effect of breast feeding on fluorosis [18,19], or any subsequent fluorosis risk on the cessation of breast feeding or the instigation of alternative/additional feeding patterns. The data obtained from parent interviews was prone to recall bias and, in some cases, information was missing or deemed too unreliable to be used, necessitating the creation of categorical variables such as the variable for feeding pattern to attempt to address this shortfall. Similarly, information on oral hygiene habits would be prone to the same recall bias or missing data and would impact on the validity of the data.

Whilst it is clear it may be possible to use fluoride content of the drinking and cooking water as an indicator in fluorosis risk assessment, the other risk factors for fluorosis cannot be ignored. The range of fluorosis presentation in this population is not remarkable in itself – some subjects have excessive exposure to fluoride resulting in severe fluorosis in a region where there is endemic fluorosis. However, the severity of fluorosis does not appear to be commensurate with this level of fluoride exposure from these sources, even when considering the likely increased intake of water (and hence fluoride) owing to climatic factors [14,20-22]. The levels of fluoride in the drinking water in this population are generally comparable to a society with fluoridated domestic water supplies such as Newcastle, England with fluoride levels adjusted to 1.0 ppm fluoride. Earlier work in Newcastle, using the same photographic scoring technique employed in the current study, revealed a prevalence of aesthetically significant fluorosis (TF3+) of 3% [23]. A crude assessment of the prevalence of aesthetically significant fluorosis (TF3+) in the current study population would be 17%. It should be stated this carries the assumptions that the study population are representative of the population as a whole. The increase in fluorosis prevalence in Newcastle was attributed to the increasing use of fluoridated dentifrices in addition to fluoridated water supplies. However, the use of fluoridated dentifrice may not be an important contributing factor in Chiang Mai where it has been demonstrated in this population the majority of children use low or non-fluoridated dentifrice. It would appear there are other contributing factors in Chiang Mai. Earlier work on subjects in Thailand failed to reconcile the fluoride intake from water with the urinary excretion of fluoride, there appeared to be an additional source of fluoride intake not being considered [24]. Later work, in a similar population in Thailand, looking at drinking water fluoride content and urinary excretion of fluoride had similar findings, but the differences could be accounted for when considering cooking water and the fluoride content of food [7].

The fluoride content of the food consumed can have an important impact on the quantity of fluoride ingested [25-27]. Rice is a staple foodstuff in the diet in Thailand and is eaten from an early age. In fact 549 subjects (98%) had reported having routinely eaten some form of rice by the age of three years. As well as being a staple in the diet rice has the capacity to contain high levels of fluoride in its cultivation, preparation and cooking. During the preparation of rice the grains are washed and then soaked for a prolonged period of time in water before cooking. If the water in which the rice is soaked is high in fluoride the resulting soaked rice can become a major source of fluoride intake [7]. It has been shown that different methods of preparation and cooking of rice can affect the final fluoride concentration [28] and even when rice is prepared with water considered to be optimally fluoridated there can be a significant effect on fluoride content and hence fluoride intake. It has been demonstrated individuals consuming a similar diet of rice and beans in an optimally fluoridated area would be subject to a dose of 0.02mmg/F/day/kg body weight – corresponding to 45% of the total intake of fluoride from foods and beverages [27]. Nevertheless, it would appear both the water used for soaking the rice and the length of time the rice is soaked have the most profound effect [7]. The use of water with a lower fluoride content such as clean rainwater for the washing and soaking process would be more appropriate than using groundwater that has high fluoride content. In addition the water used for cooking should ideally contain low levels of fluoride. Further work is needed to assess the impact of rice preparation on the overall fluoride intake and also the risk of developing fluorosis.

In this survey only the maxillary central incisors were considered in assessing the presence of fluorosis and the determination of fluorosis risk. It should be stipulated this was chosen for logistical reasons alone, as these teeth were the only teeth that could be reliably imaged and scored from the photographs for the age range of this population. It should also be stated this paper does not wish to portray the message that fluorosis risk should only be determined for the maxillary central incisors during “periods of vulnerability”. The risk of fluorosis extends across the entire dentition during the period of tooth development and is associated with the cumulative dose of fluoride over this whole time period [3].

## Conclusions

The results of this study suggest the use of the fluoride levels in current drinking and cooking water may be a reliable indicator in assessing fluorosis risk or indicating the presence or absence of aesthetically significant fluorosis in low-migratory populations. However, important risk factors such as infant feeding patterns, water used for reconstituting infant formula and oral hygiene habits should not be ignored when considering the total fluoride ingestion and fluorosis risk. Particular attention should be placed on assessing the total fluoride intake of young children in areas where there is exposure to high levels of fluoride. Further work should be conducted to explore these risk factors preferably in a prospective survey in order to assess the impact on fluorosis risk whilst assessing if there is a seasonal effect on fluoride exposure with respect to water supply. In this body of work it might be preferable to include anthropometric measurements for subjects in order to investigate fluoride dose in addition to total fluoride intake. Additional work should also be considered in assessing the risk associated with water used in the preparation of significant foodstuffs such as rice and education provided in the risks associated with the use of high fluoride water in food preparation.

## Abbreviations

ANOVA: analysis of variance; CI: confidence interval; ICOH: Intercountry Centre for Oral Health; LR: Likelihood Ratio; QLF: Quantitative light induced fluorescence; TF: Thylstrup & Fejerskov Index.

## Competing interests

RPE is an employee of a manufacturer of oral care products.

## Authors’ contributions

MGM prepared the protocol, trained staff in the use of the QLF system, carried out remote TF scoring, was involved in the analysis of data and wrote the manuscript. RPE inputted into the study design, trained staff in photographic methods, carried out remote TF scoring and inputted into the manuscript. PS & NK were local investigators responsible for interviews, water analysis and photographic examinations. HW was involved in the statistical analysis. IAP was involved in study design and manuscript preparation. All authors read and approved the final manuscript.

## Pre-publication history

The pre-publication history for this paper can be accessed here:

http://www.biomedcentral.com/1472-6831/12/16/prepub
